# Clinical education of ethicists: the role of a clinical ethics fellowship

**DOI:** 10.1186/1472-6939-5-6

**Published:** 2004-11-08

**Authors:** Paula Chidwick, Karen Faith, Dianne Godkin, Laurie Hardingham

**Affiliations:** 1 University of Toronto Joint Centre for Bioethics, Ontario, Canada; 2 Trillium Health Centre, Mississauga, Ontario, Canada; 3 William Osler Health Centre, Brampton, Ontario, Canada; 4 Sunnybrook and Women's College Health Sciences, Toronto, Ontario, Canada; 5 Centre for Clinical Ethics (a shared service of Providence Healthcare, St. Joseph's Health Centre and St. Michael's Hospital), Toronto, Ontario, Canada; 6 St. Joseph's Healthcare, London, Ontario, Canada; 7 Department of Physical Medicine and Rehabilitation, Faculty of Medicine and Dentistry, University of Western Ontario, London, Ontario

## Abstract

**Background:**

Although clinical ethicists are becoming more prevalent in healthcare settings, their required training and education have not been clearly delineated. Most agree that training and education are important, but their nature and delivery remain topics of debate. One option is through completion of a clinical ethics fellowship.

**Method:**

In this paper, the first four fellows to complete a newly developed fellowship program discuss their experiences. They describe the goals, structure, participants and activities of the fellowship. They identify key elements for succeeding as a clinical ethicist and sustaining a clinical ethics program. They critically reflect upon the challenges faced in the program.

**Results:**

The one-year fellowship provided real-time clinical opportunities that helped them to develop the necessary knowledge and skills, gain insight into the role and scope of practice of clinical ethicists and hone valuable character traits.

**Conclusion:**

The fellowship enabled each of the fellows to assume confidently and competently a position as a clinical ethicist upon completion.

## Background

Bioethics is being integrated into healthcare settings more widely and systematically than ever before. In Canada, clinical ethicists are employed in many teaching hospitals and their presence is increasing in community hospitals and long-term care facilities. Although individuals who work in the field come from diverse backgrounds with a variety of skills and training, the roles that clinical ethicists fill have some commonalities. Most clinical ethicists serve as resource persons and engage in consultation services, research, education and policy development within a healthcare setting, as well as engage in organizational ethics activities [1].

The American Society for Bioethics and Humanities has developed a set of core competencies for health care ethics consultation [2,3]. The Society suggests that ethics consultants should have skills in three general areas (assessment skills, process skills, and interpersonal skills) and knowledge in nine areas. The Society suggests that competencies can be acquired through a variety of different approaches. Although there is general agreement that education and training for clinical ethicists are important, the most effective methods of delivering that training have not yet been clearly identified [1,4-9]. The fit between the education and training students receive and the ability to assume a position in bioethics upon completion has been questioned [9,10]. There is also debate as to whether education and training programs should become more uniform and homogenous or remain heterogeneous [9,10].

A clinical ethics fellowship is perceived by some to be one of the ways in which necessary core competencies can be acquired [2]. Currently, however, clinical ethics fellowship opportunities for individuals wishing to pursue a career as a clinical ethicist are relatively limited. In a fellowship, individuals are provided with real-time clinical opportunities to help them develop necessary knowledge and skills, gain insight into the role and scope of practice of clinical ethicists and hone their character over a period of time. Specifically, clinical ethics experience may assist individuals in the development of their abilities to identify and analyze ethical problems, use reasonable clinical judgment, communicate effectively, negotiate and facilitate when there is conflict, and act as a resource for healthcare professionals who are faced with the daily challenges of delivering ethical care.

The University of Toronto Joint Centre for Bioethics (hereafter referred to as the JCB) developed and implemented a clinical ethics fellowship program to assist in meeting the identified need for clinical knowledge and skills [2,3,6,11]. In this article, a description of the fellowship is provided, including its goals, structure, participants and activities. By reflecting on their experiences, the authors, who were the program's first four participants, discuss how the clinical ethics fellowship helped prepare them to work as clinical ethicists. They identify key elements they perceive as necessary for success as a clinical ethicist and for developing an effective clinical ethics service. As well, they critically reflect upon the challenges faced as they progressed through the program.

## Method

### University of Toronto Joint Centre for Bioethics Clinical Ethics Fellowship

The JCB is a collaborating centre of the World Health Organization. It was formed in 1995 and is a partnership among the University of Toronto and its affiliated hospitals. With a membership of over 160, approximately 20 of whom work full-time in bioethics, it represents the largest multi-disciplinary group of in-hospital ethicists in Canada. Its members are widely published and actively engaged in a number of locally and nationally funded ethics research projects. In addition to the clinical ethics fellowship, the JCB offers two bioethics graduate programs.

The first two participants in the JCB's clinical ethics fellowship entered the program in July 2001. The second cohort of two fellows began the program in August 2002. (In September 2003, the program expanded to include three fellows, and in September 2004 grew to five fellows.) The primary purpose of the one-year fellowship program is to provide the necessary preparation individuals require for a smooth transition from academic and clinical education, training and experience to the position of clinical ethicist. The fellowship provides multi-site clinical ethics opportunities at both specialty and general hospitals, exposes fellows to a variety of multi-disciplinary approaches to clinical ethics, supports the work of the ethicists at the JCB's affiliated hospitals, and, lastly, expands and strengthens the network among clinical ethicists, both within the JCB and across Canada. To be eligible for the fellowship candidates must have a graduate degree in bioethics or a professional degree with significant bioethics training. Preference is given to candidates with previous exposure to clinical bioethics including consultation and teaching experiences.

### Structure of the fellowship

In the first two years the program of the program, each fellow rotated through four of the JCB's eight affiliated teaching hospitals, both specialty and general. As the number of affiliated hospitals has continued to grow, so too has the number of fellows. The fellowship was structured so that each fellow was concurrently assigned to two hospitals for a six-month period of time, averaging about two days per week on site at each hospital. A minimum of one day per week was spent working at the JCB where the fellows shared well-equipped office space. This functional arrangement promoted opportunities for collaboration, reflection and mutual support among the fellows. The fellows received a monthly stipend that was sufficient for covering basic costs of living.

## Results

### Activities in the fellowship

Throughout the year, fellows attended and actively participated in the weekly Wednesday meetings and case conferences of the JCB's Clinical Ethics Group, as well as the weekly seminars hosted by the JCB that were open to the university community and the public. The Clinical Ethics Group is comprised of the ethicists who work at the JCB's affiliated hospitals. The focus of these weekly meetings is to develop exemplary models of clinical ethics practice in diverse healthcare settings. Activities include research and practice collaborations, sharing of ideas and resources, strategic planning and policy discussions. Fellows actively participated as full members of the group in these meetings. For example, one of the projects that fellows worked on was the conception, development, and implementation of the *Project for Examining Effectiveness of Clinical Ethics (PEECE)*. PEECE was an ongoing research initiative. Its purpose was twofold: to describe the current state of affairs of clinical ethics across sites and through interviews with key stakeholders to identify benchmarks of effectiveness. Fellows participated in all aspects of the project from reviewing literature, developing a proposal, collecting and analyzing data to preparing papers for publication. Policy discussions revolved around such varied topics as sexuality in long-term care, pharmaceutical sponsorship, gift-giving in the context of professional/provider relationships and end-of-life care.

During the weekly case conferences, individual ethicists bring complex and challenging cases forward for broader consultation and review. For example, in the second year of the fellowship, a pressing clinical situation arose, with an accompanying set of complex ethical questions. This was the emergence of Severe Acute Respiratory Syndrome (SARS). The weekly case conference discussions during this time period focused on ethical issues such as the professional duty to care for and treat patients, limits of confidentiality and visitor restrictions. Among the many other cases that came to the case conference were situations of conflict around end-of-life treatment and defining futility, moral distress of staff providing care in the context of serious resource limitations, elder abuse in the community, and pregnancy termination for genetic anomalies. Fellows who were involved in the cases collaborated and co-presented with the hospital ethicist. Fellows also provided background literature, developed presentation materials and other resources for the Clinical Ethics Group on specific ethical issues as requested. In addition to providing a mechanism for acquiring broader consultation on a particularly challenging and complex case, the case conferences served as a quality assurance mechanism for the affiliated hospitals.

The weekly meetings and case conferences were a resource for the clinical ethicists and clinical ethics fellows to receive collegial support and networking opportunities. The weekly seminars featured local, national and international speakers on a wide range of topics.

Fellows were encouraged and mentored to participate in a wide range of activities at each of the affiliated hospitals. The fellows were warmly welcomed into the various institutions by the clinical ethicists, staff and patients. Participation in the preparation and delivery of formal and informal educational activities comprised the largest element of the fellowship, and occurred on at least a weekly basis and frequently more often. Educational activities included presenting at Grand Rounds on ethics topics such as clinical ethics decision-making, moral distress, and advance care planning; leading unit-based rounds on topics such as artificial hydration and nutrition at the end-of-life; facilitating brown bag lunches on topical ethics issues; teaching segments of undergraduate and graduate programs; and developing and implementing innovative curriculum for ethics committee members.

Second, case consultations were another activity in which the fellows routinely engaged. Initially, fellows participated in the preparation for case consultations and then observed the consultation process as it unfolded. They provided support for the hospital ethicists by gathering background information about the case, reviewing the relevant literature and documenting the consultation in the health record. As the Fellows progressed through the program and their skills and confidence increased, they assumed more responsibility in consultations by chairing or facilitating meetings. In addition, fellows had the opportunity mentor graduate bioethics students by including them in consultations. Throughout the fellowship, fellows received immediate feedback on the progress and outcomes of the consultations from the hospital ethicist. This debriefing opportunity was invaluable for fellows, enabling them to gain insights into the context of the case, the nature of the conflict or difficulty and the unique and recurring themes that were encountered within and across consultations. Teachable moments, individual strengths and areas for further skill and knowledge development were also identified. The number of consultations varied from one site to another, but over the course of the fellowship, each fellow was exposed to a wide variety of consultation experiences. Case consultations differed in terms of their length, from a very short 10-minute conversation to up to 6 hours in a single day with continuing follow-up over subsequent days, weeks and sometimes months.

Third, fellows participated in policy and guideline development, although these activities consumed less time than educational and consultation duties. For example, one fellow developed guidelines for the administration of blood and blood products to pediatric Jehovah's Witness patients. She then took the draft to focus groups consisting of various stakeholders both internal and external to the hospital and redrafted the guidelines based on this input.

Fourth, fellows participated in clinical ethics research and research ethics board activities. An example of such research was a chart audit conducted by a fellow to examine how consent and capacity issues were being addressed in a particular facility. Several practice concerns were identified and subsequently a facility-wide educational program was implemented. In addition, the fellows engaged in a variety of other scholarly activities including writing, presenting and publishing on ethics-related topics in a variety of forums, which allowed the fellows to develop a comprehensive understanding of a wide variety of strategies for building a sustainable, integrated and accountable ethics program. These experiences, which built professional knowledge, skill and confidence, laid the foundation for the fellows in developing their professional identity as clinical ethicists. Observing the hospital ethicists in action, the fellows realized that these clinical ethics roles were developed over time and with effort. This helped to shape realistic goals and expectations for the early phase of a clinical ethics career.

### The first fellows

In July 2001, Paula Chidwick and Laurie Hardingham were the first fellows accepted into the JCB's clinical ethics fellowship program. Dr. Chidwick holds a PhD in Philosophy and prior to entering the fellowship program completed an ethics internship at Sunnybrook and Women's College Health Sciences JCB. She has taught bioethics at the University of Toronto. Laurie Hardingham is a registered nurse who has worked in a variety of healthcare settings. She has comprehensive academic education in philosophy, completing a Masters in Philosophy and doctoral course work in philosophy. She has taught philosophy and ethics at the University of Calgary and Mt. Royal College, as well as planned and coordinated the Provincial Health Ethics Network in Alberta.

Karen Faith and Dianne Godkin were selected for the 2002/2003 clinical ethics fellowship program. Karen Faith completed a Masters in Science majoring in bioethics through the Collaborative Program at the Joint Centre for Bioethics and the Institute of Medical Sciences, University of Toronto. After completing her degree in bioethics, Ms. Faith was a part-time ethics consultant to several healthcare organizations. Previously, she was a social worker who worked in the area of mental health. Ms. Faith has taught at York University, Seneca College and Centennial College in Toronto. Just prior to beginning the clinical ethics fellowship, Dianne Godkin completed a PhD in Nursing. During her doctoral studies she focused on ethics and gerontology, particularly in the areas of end-of-life decision-making and advance care planning. While studying at the University of Alberta, she taught an interdisciplinary graduate course in health ethics and was an observer on a healthcare ethics committee. The objectives that the fellows set out to accomplish during the fellowship included gaining expertise in the clinical consultation process, further developing their teaching and researching skills, increasing their confidence in working through difficult ethical situations as they unfold and expanding their multi-disciplinary network of contacts.

## Discussion

### Preparing fellows to work as clinical ethicists

The fellowship helped prepare the fellows to make the transition to clinical ethicists by providing real-time clinical opportunities. Although there were opportunities to attend lectures, seminars and conferences and to participate in research projects and the activities of research ethics boards, the focus of this fellowship was clinical practice.

### "Real-time" clinical opportunities

Generally, bioethics education is largely theoretical, focusing on academic course work in philosophy and ethics, as well as other disciplines, at the graduate level. Practical clinical experiences for individuals wishing to pursue a career as a clinical ethicist have been very limited historically and offered only sporadically. In this fellowship, ethical challenges unfold and are addressed within the day-to-day experiences of hospital life. Although hypothetical or retrospective cases studied in the classroom are useful in applying theory to clinical cases, the value of experiential knowledge gained when cases are encountered in the here and now involving real people with tangible consequences cannot be overstated. One fellow recalls a case involving a family having a very difficult time coming to terms with the imminent death of a loved one. The family was adamant that "everything be done", a phrase that often is bandied about in these sorts of discussions and requires considerable exploration. In this case, "everything" was defined by the family to include CPR and admission to intensive care. The fellow attended a meeting with the family and the healthcare team to discuss the plan of care, but the patient, although capable, was too ill to attend. The fellow had not met the patient. The description of the patient by the healthcare team was completely different from that given by the family. The fellow was uncomfortable with the decisions made without directly hearing the patient's voice. It was not until the fellow met with the patient and the physician alone, that she began to understand the situation. Seeing the physical frailty, but clear thinking and comprehension of the patient fuelled her wish to see that the patient received the care that she desired. It mattered what decisions were made, the situation was no longer hypothetical, but was real and the stakes were high.

Through their daily work and interactions with staff in the various hospitals, the fellows became familiar with the fast-paced clinical environment and culture, the healthcare providers' values and practices and the complexity and diversity of ethical issues. Given the unpredictability of when consultation requests would surface, fellows found themselves needing to be flexible and accommodating, often leaving writing or research activities to respond to requests for consultation. Fellows could be called to the intensive care unit, coronary care unit, emergency department, or hospital boardroom at any time and some of these consultations required an immediate response. Consultations of a less emergent nature were scheduled for a later time and often included meetings with the healthcare team, families and patients. Fellows carried a pager so that they could be reached immediately.

Other learning opportunities included the following: developing and implementing an ethics program through participation in strategic planning activities; raising the profile of ethics in a hospital using a variety of networking, public relations and communication strategies; reaching out to those who questioned the value of ethics programs by establishing an ongoing presence on units that were struggling with a particular ethics issue; building trust and establishing credibility with healthcare professionals by recognizing, understanding and responding first to their most urgent needs; identifying opinion leaders in the organization and integrating them into the ethics program; building bridges with senior management; and supporting the work of ethics committees as well as other hospital committees

### Skill development

Throughout the year, the fellows each worked with a number of clinical ethicists with varied approaches, backgrounds, training and expertise. As a result there were numerous and ongoing opportunities to develop a multiplicity of skills. Through the observation and mentoring of the clinical ethicists, fellows honed their mediation, communication and negotiating skills. They developed political, practical and conflict resolution skills in both observing and responding to conflicts pertaining to patient care decisions They learned to use wisdom or judgment, particularly in establishing credibility, gaining trust and responding to challenges regarding their role and duties. For example, when a fellow witnessed a clinical ethicist's role being challenged by a senior hospital staff member, the clinical ethicist modeled a respectful but assertive approach, demonstrating both good judgment and clarity of purpose. They acquired skills in the recognition, prevention and management of moral distress and moral residue. Many of the clinical ethicists shared personal experiences of morally distressing situations and modeled the need for broad consultation through the JCB consultation group and debriefing with colleagues as a way to cope with stress. The development of this last skill has proven invaluable as the role that moral distress and residue play in the clinical setting becomes increasingly acknowledged and better understood [13,16].

The skills that were nurtured and developed during the fellowship mirror the ethical assessment skills, process skills and interpersonal skills that have been identified as core competencies for ethics consultation [2,3] As the fellows moved through the program, they received ongoing critique of their skills. They participated in educational and practice activities to support their skill development (for example, conflict negotiation workshops).

### Insights into the role and scope of practice of clinical ethicists

The fellows observed that the scope and practice of the clinical ethicists included four primary areas of focus: building capacity, acting as a resource, organizational ethics and scholarly work. The goals of capacity building within the organization included promoting ethical sensitivity and discernment, increasing ethics knowledge and skills and enhancing ethical behavior in the delivery of healthcare. This was accomplished through formal and informal educational activities, committee work, consultations and daily interactions with staff. As a resource, clinical ethicists were called upon to do ethics consultations, provide information and share expertise in various areas of ethical concern. Clinical ethicists' organizational ethics activities were diverse and included the development of policy, guidelines and procedures, collaborative initiatives with other departments and professionals and strategic planning. As well, all of the clinical ethicists were engaged in scholarly activities such as research, writing and publishing, presenting at conferences and teaching at universities and colleges.

As a result of working with clinical ethicists in a variety of healthcare settings with different educational backgrounds, the fellowship experience offered a broad perspective on the role and scope of clinical ethics practice. Because clinical ethics is a relatively young field that continues to evolve and define itself [6,7,17,18], seeing and working with clinical ethicists in action, demonstrating their skills and knowledge, was instructive and assisted the fellows in developing their own professional identity and understanding of what an ethicist's role and responsibilities were and were not in the healthcare setting. The fellows learned that common misperceptions of the clinical ethicist's role included that of moral expert, judge of right and wrong, legal expert, risk manager, ethics police, ombudsperson, locus of ethics for the institution and final decision-maker [19,20].

### Character development

By observing and participating with the clinical ethicists in their daily activities the fellows identified certain important character traits for this role, such as humility, respect for others, self-knowledge, self-awareness and courage. Although other character traits were also observed, the fellows agreed that these particular traits were both necessary and desirable and thus worthy of emulation in their own practice.

The fellows observed that clinical ethicists who modeled humility recognized that their role was neither that of judge nor moral expert, but as a member of the team who was able to engage in a collegial process of deliberation and ethical decision-making. As well, with humility came the recognition that one ethicist cannot be knowledgeable in all areas and that it was essential to build up a network of colleagues from different educational backgrounds with whom to consult. Similar traits such as self-knowledge and self-awareness involved the ability of the ethicist to recognize his or her strengths and limitations. The extent to which the ethicist demonstrated self-knowledge and self-awareness influenced their own self-care practices and ability to manage work demands and work related-stress and thus avoid burnout. Fellows observed ethicists maintaining an attitude of respect toward the opinions of all concerned parties; they ensured that each individual's voice was heard and his or her perspective considered. When clinical ethicists upheld an ethical position in the face of considerable opposition the fellows concluded that ethicists modeled courage. The traits deemed important by the fellows reflect many of the character traits that are considered to be prerequisites to successful healthcare ethics consultation [2,11]. Further contemplation on these traits by the fellows raised their own level of self-awareness and their desire and ability to integrate and exhibit these traits in their daily practice.

### Key elements for success

Through their fellowship experiences in a variety of ethics programs at differing stages of development, the fellows recognized certain elements that appeared to contribute to an effective clinical ethics program. First, a clinical ethics program needs to be integrated throughout the organization. Integration was key in building capacity from bedside to boardroom and dispelling myths about the role of ethics and ethics programs. Embedding ethical considerations into all aspects of decision-making is achieved through an understanding of how ethics can be a resource for the staff when they face ethical dilemmas. Indicators of a well-integrated ethics program included a clear understanding of the program by staff, visibility within the organizational structure and accessibility of the ethicist to staff, patients and families.

Second, a sustainable ethics program requires organizational support and a commitment through the provision of a dedicated budget for ethics including administrative support, adequate physical space and resources, as well as support for continued education. Organizational commitment can be demonstrated through a clearly defined and stable reporting structure and the clinical ethicist's participation in decision-making at the management level. Such organizational commitment allows the ethicist the resources and time to provide the services that support excellence in patient care and to help staff when faced with ethical issues. The clinical ethicist needs to have clear goals and parameters for the work and establish reasonable expectations in order to provide an effective service, reducing ethicists' moral distress and burnout.

Third, clinical ethicists cannot work in isolation and need the support of a network of colleagues both within and outside of the field of ethics, especially when confronted with complex or unusual cases in new and emerging areas. One of the roles of clinical ethicists is to act at the same time as both trusted organizational insider and as an objective neutral outsider. Clinical ethicists are best able to succeed in this capacity when they develop collaborative relationships with other service providers in the healthcare settings for example, risk management, pastoral care and social work. Fellows observed that this network of support included the JCB clinical ethics group as well as key professionals knowledgeable in areas of bioethics relevant to the specialized areas of health care. For example, one clinical ethicist had particular expertise in pediatric settings and was called upon often by colleagues when an ethical challenge concerned the care of neonates or children.

Fourth, the clinical ethicist's ability to see beyond the initial presenting problem was a crucial skill in the case consultation process. As the clinical ethicist entered into the situation the scope of inquiry often broadened and new and larger, and sometimes quite different, questions emerged. For example, when called in by staff for a consultation, the fellows often observed that upon discussion with the patient or family a different problem was brought to light. Fellows observed that ethicists that kept the dynamic nature of the consultation in mind usually had more successful consults.

### Critical reflections

Christine Harrison challenges those engaged in bioethics to consider what "bioethics *is*" before contemplating its future [6]. The clinical ethics fellowship assisted the fellows in developing their own understanding of what clinical ethics *is *and the clinical ethicist's role, as well as acquiring the necessary knowledge, skills and character traits. The one-year practical learning experience in clinical ethics was perceived by the fellows as an excellent way for them to begin to understand what it means to be a clinical ethicist and to develop core competencies to succeed in that role. However, as the field is evolving quickly with new issues emerging, sometimes quite unexpectedly, it is unlikely that one would ever feel fully prepared to independently step into the position of clinical ethicist. The fellows in the second cohort learned this lesson first-hand, when Severe Acute Respiratory Syndrome (SARS) struck Toronto and dramatically transformed the work environment in the hospitals in which they served [12]. Rotations were in six-month segments with a shared work week between two hospitals, but due to SARS precautions which prohibited people from traveling between sites, fellows needed to limit their work to one hospital. Indeed, some of the fellows were not allowed into particular hospitals until infection control restrictions were lifted and were forced to continue their work from home as best they could. Even prior to SARS, fellows found that the disparate geographic location of multiple work settings made availability for consults difficult at times. Subsequently, full-time three-month block placements for fellows have been implemented at some hospital sites rather than the split workweek.

After the first year of the program, a position became available for a one-year senior clinical ethics fellowship. Laurie Hardingham accepted that position, and during the senior fellowship year, she worked in one teaching hospital, concentrating on ethics consultations, increasing educational opportunities for staff and strengthening the clinical ethics program in that hospital. She was also available to mentor and advise the new first year fellows, supporting the fellowship program. The senior fellowship allowed her to develop a greater understanding of how to integrate ethics throughout an organization and develop the ability to more effectively utilize organizational structures and resources in the clinical ethics program.

The hospital ethicists that the fellows assisted had many organizational commitments, were involved in numerous projects and could be called upon at a moment's notice for consultations. As the areas of focus for clinical ethics services varied significantly between hospital settings, fellows were required to review and research literature on many complex and different ethical, clinical and legal topics. To meet the demands of working in a fast-paced healthcare environment with rapidly changing needs, fellows were also faced with the challenges of being available, flexible and accommodating. Being introduced to several hospital settings at the beginning of each rotation presented the fellows with the additional tasks of quickly familiarizing themselves with and acclimatizing to new organizational rules and procedures, staff and institutional cultures. Being a fellow also brought in practical considerations such as taking leave from previously held positions, adjusting to a considerable reduction in pay and relocating to Toronto.

The fellows were exposed to stylistic and theoretical differences in the way clinical ethics was practiced when working with ethicists who entered the field through diverse academic and clinical backgrounds. The potential does exist for such differences to become a barrier to learning and building trust within the clinical ethicist/fellow relationship and the fellows who experienced this learned about developing working relationships with ethicists whose priorities differed. For example, when the hospital ethicists also had responsibilities as physicians or nurses in addition to clinical ethics responsibilities, the perspectives could differ on which activities receive attention first. Therefore, it is essential that support be made available in the form of advocacy and mediation for the fellows should such a conflict arise. In this program, such support is available through the program's coordinator at the Joint Centre for Bioethics.

## Conclusions

Not unlike the field of bioethics itself, the Joint Centre for Bioethics Clinical Ethics Fellowship program is evolving with each successive year and will ultimately be judged by how well graduates are integrated into the healthcare community and the contributions they make to the field. The fellows concur that none of them would have felt sufficiently prepared to take on the considerable responsibilities, complex role demands and inevitable moral distress that are inherent in the position of clinical ethicist without the fellowship. Participation in the fellowship was instrumental in helping the fellows develop the necessary clinical ethics skills, knowledge and character traits required for them to assume a role as a clinical ethicist in a healthcare setting. As well, through the fellowship, they cultivated a support network for the future.

Since completing the fellowship, each of the first four fellows has obtained a position as a clinical ethicist in a healthcare setting. Because of their fellowship experiences, they embark on their new careers with a realistic picture of clinical ethics, demonstrated core competencies and a strong network of ethics support and expertise to draw upon in the future. Although other educational models for clinical ethicists exist, a clinical ethics fellowship that is applicable to individuals from a variety of backgrounds (i.e., not limited to clinicians or philosophers only) appears to be a viable educational option and one that ought to be further developed and more formally evaluated.

## List of abbreviations used

JCB – The University of Toronto Joint Centre for Bioethics

SARS – Severe Acute Respiratory Syndrome

## Competing interests

The author(s) declare that they have no competing interests.

## Authors' contributions

PC contributed substantially to the conception and design, analysis and interpretation of data, drafting of the article and revising it critically and gave final approval for the version to be published.

KF contributed substantially to the conception and design, analysis and interpretation of data, drafting of the article and revising it critically and gave final approval for the version to be published.

DG contributed substantially to the conception and design, analysis and interpretation of data, drafting of the article and revising it critically and gave final approval for the version to be published.

LH contributed substantially to the conception and design, analysis and interpretation of data, drafting of the article and revising it critically and gave final approval for the version to be published.

## Pre-publication history

The pre-publication history for this paper can be accessed here:


